# Transcriptional activation by MafR, a global regulator of *Enterococcus faecalis*

**DOI:** 10.1038/s41598-019-42484-4

**Published:** 2019-04-16

**Authors:** Sofía Ruiz-Cruz, Ana Moreno-Blanco, Manuel Espinosa, Alicia Bravo

**Affiliations:** Centro de Investigaciones Biológicas, Consejo Superior de Investigaciones Científicas, Madrid, Spain

## Abstract

Proteins that act as global transcriptional regulators play key roles in bacterial adaptation to new niches. These proteins recognize multiple DNA sites across the bacterial genome by different mechanisms. *Enterococcus faecalis* is able to survive in various niches of the human host, either as a commensal or as a leading cause of serious infections. Nonetheless, the regulatory pathways involved in its adaptive responses remain poorly understood. We reported previously that the MafR protein of *E. faecalis* causes genome-wide changes in the transcriptome. Here we demonstrate that MafR functions as a transcription activator. *In vivo*, MafR increased the activity of the *P12294* and *P11486* promoters and also the transcription levels of the two genes controlled by those promoters. These genes are predicted to encode a calcium-transporting P-type ATPase and a QueT transporter family protein, respectively. Thus, MafR could have a regulatory role in calcium homeostasis and queuosine synthesis. Furthermore, MafR recognized *in vitro* specific DNA sites that overlap the −35 element of each target promoter. The MafR binding sites exhibit a low sequence identity, suggesting that MafR uses a shape readout mechanism to achieve DNA-binding specificity.

## Introduction

Global transcriptional regulators play crucial roles during bacterial adaptation to specific niches. They activate and/or repress the transcription of multiple genes and, therefore, make possible to rapidly adjust the gene expression pattern to new environmental situations. *Enterococcus faecalis* is usually found as a harmless commensal in the human gastrointestinal tract. However, this Gram-positive bacterium is able to colonize other niches of the human host and cause a variety of life-threatening infections, such as urinary tract infections, endocarditis or bacteraemia^1–3^. Despite the pathogenic potential of *E. faecalis*, our understanding of the regulatory circuits involved in its adaptive responses is still very limited.

The MafR protein (482 amino acids) of *E. faecalis* is highly conserved among the strains whose genomes have been totally or partially sequenced^4^. Genome-wide microarray assays showed that MafR is involved in global regulation of gene expression^5^. In such experiments, the transcriptional profiles of strains OG1RF (wild-type) and OG1RF∆*mafR* (*mafR* deletion mutant) were compared, demonstrating that MafR activates, directly or indirectly, the expression of at least 87 genes. Many of them are organized in operons and encode proteins involved in the utilization of carbon sources (*e.g*. mannitol, glycerol, gluconate, maltose and citrate). Furthermore, compared to OG1RF, the OG1RF∆*mafR* strain was shown to induce a lower degree of inflammation in the peritoneal cavity of mice. Because of these findings, we proposed that MafR could facilitate the growth of *E. faecalis* in particular human niches and, consequently, could contribute to its potential virulence^5^.

Different protein-DNA recognition mechanisms have been characterized. In some cases, proteins recognize a sequence-dependent DNA shape (shape readout mechanism) rather than the unique chemical signatures of the DNA bases (base readout mechanism)^6,7^. MafR is a new member of the Mga/AtxA family of global transcriptional regulators^4,5^. This family includes AtxA from *Bacillus anthracis*, Mga*Spn* from *Streptococcus pneumoniae*, and Mga from *S. pyogenes*. Like these three regulatory proteins^8^, MafR has two putative helix-turn-helix DNA-binding motifs within the N-terminal region, the so-called HTH_Mga (residues 11–69) and Mga (residues 76–164) motifs^5^. In the Mga regulator, both motifs were found to be required for DNA-binding and transcriptional activation^9,10^. *In vitro* protein-DNA interaction studies have shown that MafR binds to linear double-stranded DNAs with little or no sequence specificity. Furthermore, MafR was able to generate multimeric complexes on linear double-stranded DNAs^4^. Similar DNA-binding properties have been described for the pneumococcal Mga*Spn* regulator. Mga*Spn* has a preference for AT-rich DNA sites, as well as a high affinity for a naturally occurring curved DNA^11–13^. On DNA fragments that contain the promoter of the *mafR* gene (*Pma* promoter), MafR recognizes a potentially curved DNA region, which is located upstream of the promoter (positions −69 to −104)^4^. We hypothesized that MafR, and most likely the regulators of the Mga/AtxA family, recognizes structural features in its target DNAs rather than specific nucleotide sequences^4^. Nevertheless, verification of this hypothesis requires the identification of additional MafR binding sites across the bacterial genome.

A further DNA microarray assay using an OG1RF∆*mafR* derivative that overproduces MafR (plasmid-encoded MafR) allowed us to identify two new potential MafR target genes: *OG1RF_12294* and *OG1RF_11486*. In the presence of plasmid-encoded MafR, the highest increase in gene expression corresponded to both genes (our unpublished results). In this manuscript, we addressed the validation of such a finding by *in vivo* and *in vitro* approaches. Gene *OG1RF_12294* encodes a putative phosphorylated intermediate-type ATPase (P-type ATPase) transporter, which could contribute to maintain calcium homeostasis. Gene *OG1RF_11486* encodes a putative QueT transporter family protein, which could be involved in uptake of a queuosine biosynthetic intermediate. Here we demonstrate that MafR activates directly the transcription of both genes by binding to a specific DNA site overlapping the core promoter. Such sites exhibit a low sequence identity. This study shows, for the first time, that MafR functions as a transcription activator. Moreover, it supports that MafR might recognize particular DNA shapes.

## Results

### Transcription of *mafR* under laboratory conditions

The genome of the *E. faecalis* strain OG1RF has been totally sequenced (GenBank CP002621.1)^14^. By quantitative RT-PCR (qRT-PCR) assays and using the comparative *C*_T_ method^15^, we determined the relative expression of the regulatory *mafR* gene (locus_tag *OG1RF_12293*) in cells grown under laboratory conditions (Brain Heart Infusion (BHI) broth, 37 °C, without aeration) to both logarithmic and stationary phases. Transcription of *mafR* was found to be higher at logarithmic phase. Compared to stationary phase, the fold change (log_2_FC) in *mafR* expression was ∼4. Therefore, all the experiments shown in this work were performed at the logarithmic growth phase.

### Gene *OG1RF_12294* encodes a putative P-type ATPase cation transporter

**Figure 1:**
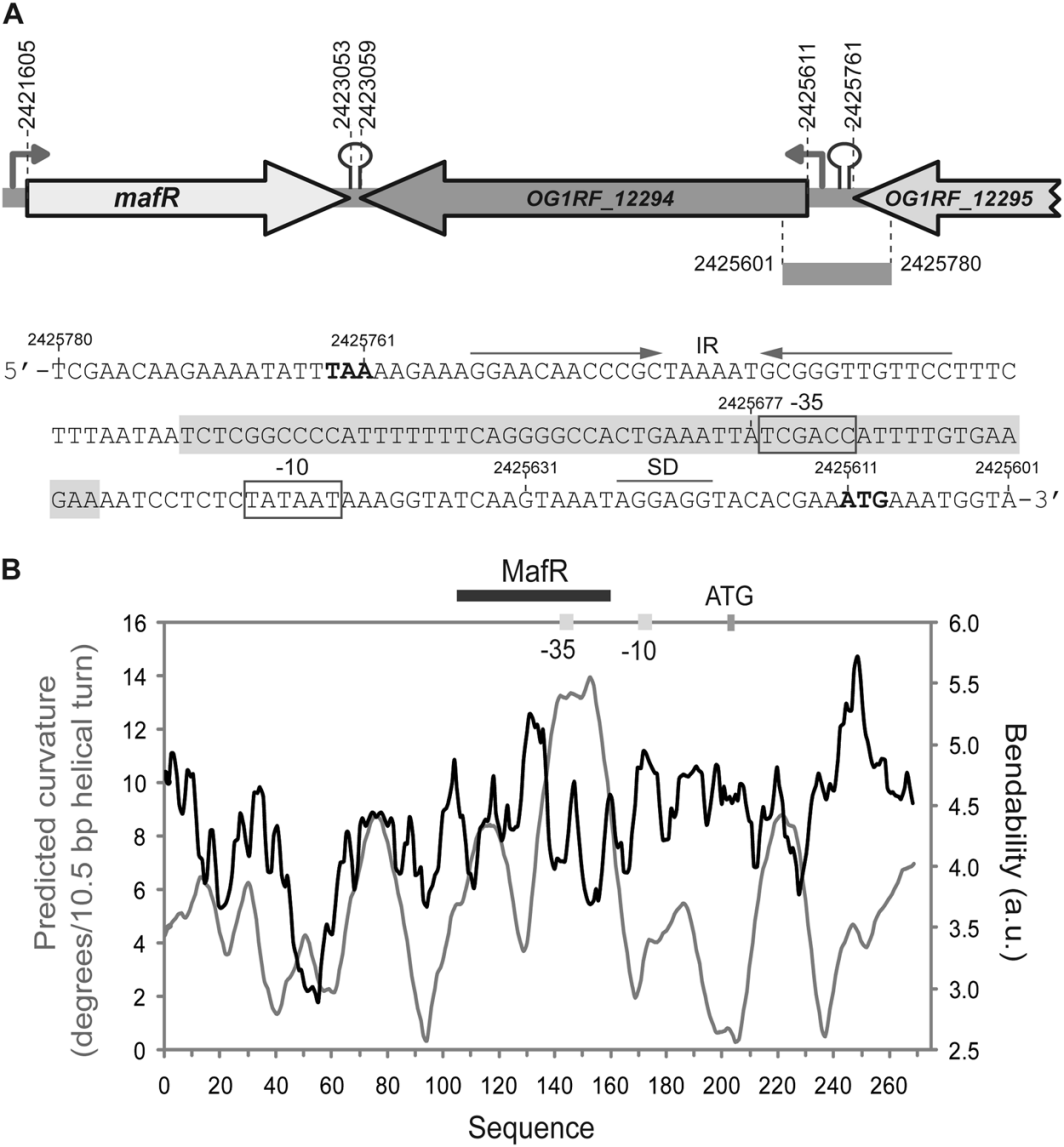
Relevant features of the *P12294* promoter region. (**A**) Genetic organization of the chromosome region that contains *OG1RF_12294*. Coordinates of the translation start and stop codons are indicated. Stem-loop elements represent potential transcriptional terminators. Arrows upstream of the genes represent promoters. The nucleotide sequence of the region spanning coordinates 2425780 to 2425601 is shown. The stop codon (TAA) of *OG1RF_12295* and the start codon (ATG) of *OG1RF_12294* are indicated in boldface letters. IR: inverted-repeat. SD: Shine-Dalgarno sequence. The main sequence elements (−35 box and −10 box) of the *P12294* promoter are indicated. The MafR binding site defined in this work is shown (shadowed box). Genes *OG1RF_12294* and *OG1RF_12295* correspond to genes *EF3014* and *EF3015* in *E. faecalis* strain V583. (**B**) Bendability/curvature propensity plot of the region spanning coordinates 2425817 to 2425548. The location of the *P12294* core promoter, the start codon of *OG1RF_12294* and the MafR binding site are indicated.

P-type ATPases constitute a large superfamily of cation and lipid pumps that use ATP hydrolysis for energy. They are integral, multispanning membrane proteins that are found in bacteria and in a number of eukaryotic plasma membranes and organelles^16^. The enterococcal *OG1RF_12294* gene, which is adjacent to *mafR* (Fig. 1A), encodes a putative P-type ATPase cation transporter. Such a gene has been annotated as *pmr1* (GeneID: 12289043) because it encodes a protein (850 amino acids) that has sequence similarity (∼52%) to eukaryotic PMR1 (plasma membrane ATPase related) P-type ATPases (Supplementary Table S1). Some PMR1-type pumps are able to transport calcium, as well as manganese, into the Golgi apparatus^17–19^.

In addition to OG1RF_12294, the OG1RF genome encodes two putative calcium-transporting ATPases: OG1RF_10600 and OG1RF_11602 (Supplementary Table S2). Using the BLASTP protein sequence alignment program^20^, we found that OG1RF_12294 has sequence similarity (∼53–56%) to both ATPases (Supplementary Table S1). Furthermore, OG1RF_12294 has sequence similarity (∼53–56%) to several prokaryotic proteins characterized as calcium P-type ATPases (Supplementary Table S1)^21–25^. Thus, protein OG1RF_12294 might contribute to maintain calcium homeostasis in enterococcal cells.

### MafR influences positively the transcription of *OG1RF_12294*

To analyse whether MafR regulates the expression of the *OG1RF_12294* gene, we determined its relative expression in OG1RF (wild-type) and OG1RFΔ*mafR* (deletion mutant) by qRT-PCR. The log_2_FC in *OG1RF_12294* expression due to the presence of MafR was ∼3, indicating that MafR has a positive effect on the transcription of such a gene. This conclusion was further confirmed by increasing the intracellular level of MafR. Specifically, we determined the relative expression of *OG1RF_12294* in two strains: OG1RFΔ*mafR* harbouring pDLF (absence of MafR) and OG1RFΔ*mafR* harbouring pDLF*mafR* (plasmid-encoded MafR). In addition, we determined the relative expression of the *OG1RF_10600* and *OG1RF_11602* genes, which encode putative calcium-transporting ATPases (Supplementary Table S1). In the presence of plasmid-encoded MafR, only transcription of *OG1RF_12294* was increased (log_2_FC ∼4). Thus, MafR influences positively and specifically the transcription of the *OG1RF_12294* gene.

### MafR activates the *P12294* promoter *in vivo*

In the OG1RF genome^14^, the ATG codon at coordinate 2425611 is likely the translation start site of the *OG1RF_12294* gene (Fig. 1A). It is preceded by a putative ribosome binding site sequence (AGGAGG). Upstream of such a sequence there is a putative promoter (here named *P12294*) that has a canonical −10 element (**TATAAT**) but lacks a potential −35 element (consensus **TTGACA**) at the optimal length of 17 nucleotides. Nevertheless, there is a near-consensus −35 element (**T**C**GAC**C) at the suboptimal spacer length of 22 nucleotides. These features suggested that promoter *P12294* could be recognized by a σ factor similar to the *Escherichia coli* σ^70^ and that its activity could be enhanced by regulatory proteins. Sequence analysis of the region located between the TAA stop codon of the *OG1RF_12295* gene (coordinate 2425761) and the *P12294* promoter revealed the existence of an inverted-repeat (IR) that may function as a Rho-independent transcriptional terminator (Fig. 1A).

**Figure 2:**
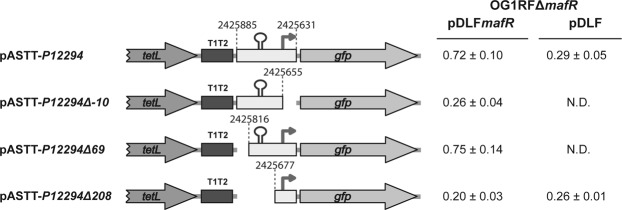
Effect of plasmid-encoded MafR on the activity of the *P12294* promoter. Four regions from the OG1RF chromosome were inserted independently into the *Sac*I site of pASTT. The coordinates of such regions are indicated. Gene *tetL*: tetracycline resistance determinant. Gene *gfp*: green fluorescent protein. The T1T2 box represents the tandem transcriptional terminators T1 and T2 of the *Escherichia coli rrnB* rRNA operon. The stem-loop element represents the inverted-repeat located upstream of the *P12294* promoter (see Fig. 1A). The arrow represents the canonical −10 element of the *P12294* promoter. The intensity of fluorescence (arbitrary units) corresponds to 0.8 ml of culture (OD_650_ = 0.4). In each case, three independent cultures were analysed. N.D.: non-determined.

To characterize the *P12294* promoter, a 255-bp DNA fragment (coordinates 2425885 to 2425631) (Fig. 2) was inserted into the pASTT promoter-probe vector, which is based on the *gfp* reporter gene. The recombinant plasmid (pASTT-*P12294*) was first introduced into OG1RF and OG1RFΔ*mafR*. In these strains, the expression of *gfp* (0.32 ± 0.02 and 0.26 ± 0.04 units, respectively) was similar to the basal level (OG1RF harbouring pASTT; 0.38 ± 0.02 units). Different results were obtained when pASTT-*P12294* was introduced into OG1RFΔ*mafR* harbouring either pDLF or pDLF*mafR* (plasmid-encoded MafR) (Fig. 2). The expression of *gfp* was ∼2.5-fold higher in the presence of plasmid-encoded MafR. This result indicated that the 255-bp DNA fragment contains a MafR-dependent promoter activity. Removal of the −10 element of the *P12294* promoter resulted in loss of such an activity (plasmid pASTT-*P12294Δ-10*). A further deletion analysis allowed us to conclude that the 186-bp region between coordinates 2425816 and 2425631 contains both the *P12294* promoter and the site required for its activation by MafR (plasmids pASTT-*P12294Δ69* and pASTT-*P12294Δ208*) (Fig. 2).

### MafR binds to the *P12294* promoter region *in vitro*

**Figure 3:**
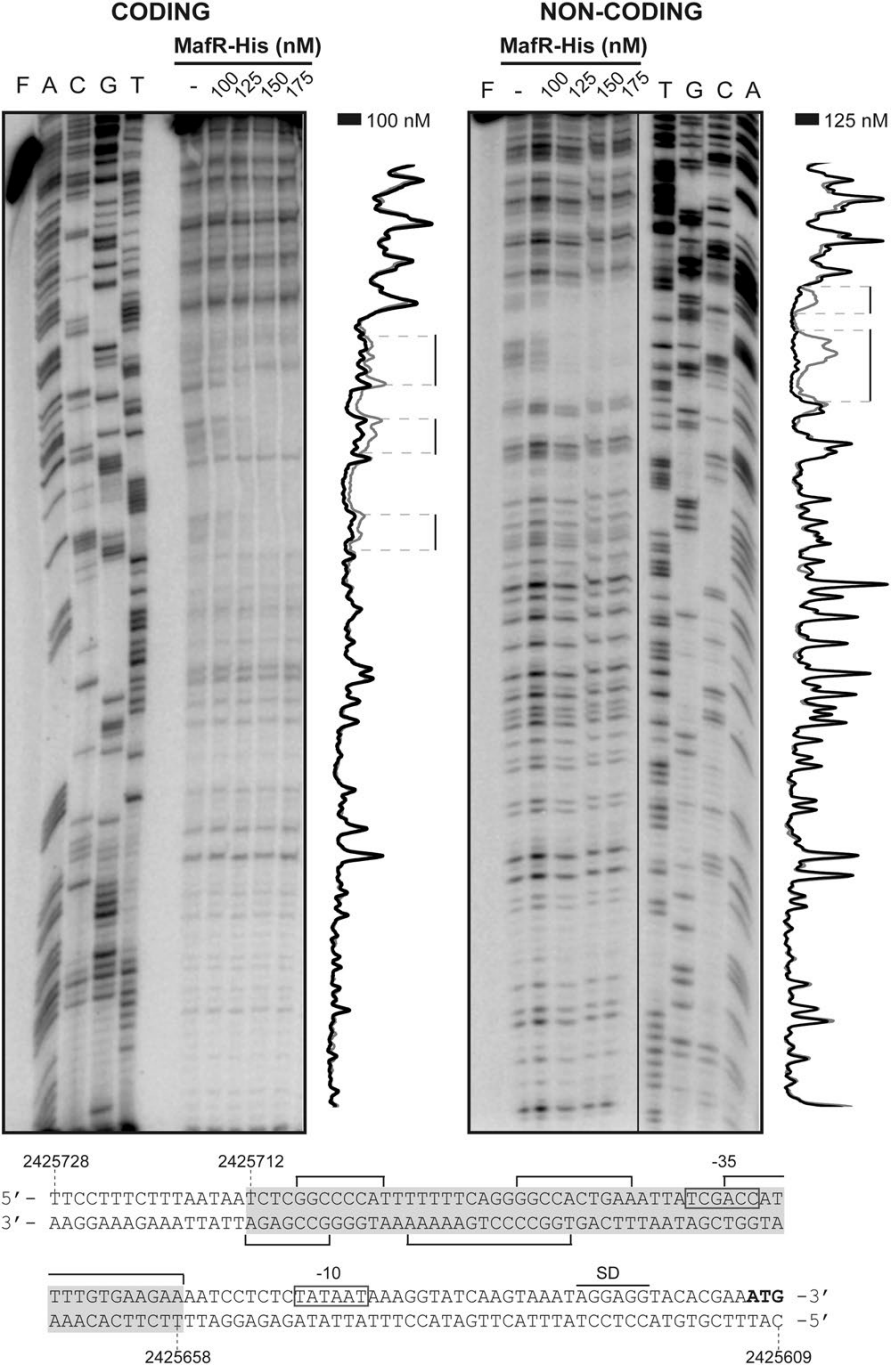
DNase I footprints of complexes formed by MafR-His on the 270-bp DNA fragment that contains the *P12294* promoter. ^32^P-labelled DNA (2 nM) was incubated with the indicated concentrations of MafR-His and then it was digested with DNase I. Non-digested DNA (F) and dideoxy-mediated chain termination sequencing reactions (A, C, G, T) were run in the same gel. All the lanes displayed came from the same gel (delineation with dividing lines). Densitometer scans corresponding to free DNA (grey line) and DNA with protein (black line) are shown. The nucleotide sequence of the region spanning coordinates 2425728 to 2425609 is shown. The −35 and −10 boxes of the *P12294* promoter are indicated. SD: Shine-Dalgarno sequence. Brackets indicate regions protected against DNase I digestion. The site recognized by MafR-His (coordinates 2425712-2425658) is indicated with a grey box.

To investigate whether MafR activates directly the expression of the *OG1RF_12294* gene, we performed DNase I footprinting experiments. We used a His-tagged MafR protein (MafR-His) and a 270-bp DNA fragment (coordinates 2425817 to 2425548). This fragment contains the *P12294* promoter and the site required for its activation by MafR *in vivo* (Fig. 2). The presence of a His-tag at the C-terminal end of MafR does not affect its DNA-binding properties^4^. The 270-bp DNA fragment was radioactively labelled either at the 5′-end of the coding strand or at the 5′-end of the non-coding strand (Fig. 3). On the coding strand and at 100 nM of MafR-His, protections against DNase I digestion were observed within the region spanning coordinates 2425708 and 2425658. On the non-coding strand and at 125 nM of MafR-His, diminished cleavages were observed between coordinates 2425712 and 2425686. Thus, MafR-His recognizes a site overlapping the −35 element of the *P12294* promoter (Fig. 3). This result allowed us to conclude that MafR activates directly the transcription of the *OG1RF_12294* gene.

Figure 1B shows the bendability/curvature propensity plot of the 270-bp DNA fragment according to the bend.it program^26^. The profile contains an intrinsic curvature of high magnitude (~13 degrees per helical turn), which is adjacent to the MafR binding site. In addition, the site recognized by MafR contains a region of potential bendability (~5.2 units).

### Gene *OG1RF_11486* encodes a putative QueT transporter family protein

Energy-coupling factor (ECF) transporters are a family of ATP-binding cassette (ABC) transporters that are responsible for the uptake of essential micronutrients in prokaryotes. They consist of a membrane-embedded S-component that provides substrate specificity and a three-subunit ECF module that couples ATP hydrolysis to transport. In the so-called group II ECF transporters, different S-components share the same ECF module. Furthermore, the S-component genes are not located in the same operon as the genes for the ECF module^27–29^.

The enterococcal *OG1RF_11486* gene encodes a putative QueT transporter family protein (GenBank AEA94173.1). Proteins identical to OG1RF_11486 (173 residues) are encoded by *Mycobacterium abscessus* (CPW17925.1), *Listeria monocytogenes* (CWW42654.1; 172 up to 173 residues are identical) and *S. agalactiae* (KLL29182.1). In the two former bacteria, the corresponding protein has been annotated as queuosine precursor ECF transporter S-component QueT. Therefore, protein OG1RF_11486 could be involved in the uptake of a queuosine biosynthetic intermediate. Using the BLASTP program^20^, we found that the OG1RF genome encodes an additional QueT transporter family protein (OG1RF_12031; 168 residues; AEA94718.1). It has 55% of similarity to the OG1RF_11486 protein.

### MafR activates the *P11486* promoter *in vivo*

By qRT-PCR assays, we found that MafR has a positive effect on the transcription of *OG1RF_11486*. Compared to strain OG1RFΔ*mafR*, the relative expression of *OG1RF_11486* was slightly higher in strain OG1RF (log_2_FC ∼0.9). Moreover, the relative expression of *OG1RF_11486* was higher in strain OG1RFΔ*mafR* harbouring pDLF*mafR* (plasmid-encoded MafR) than in strain OG1RFΔ*mafR* harbouring pDLF (log_2_FC ∼2.4).

**Figure 4:**
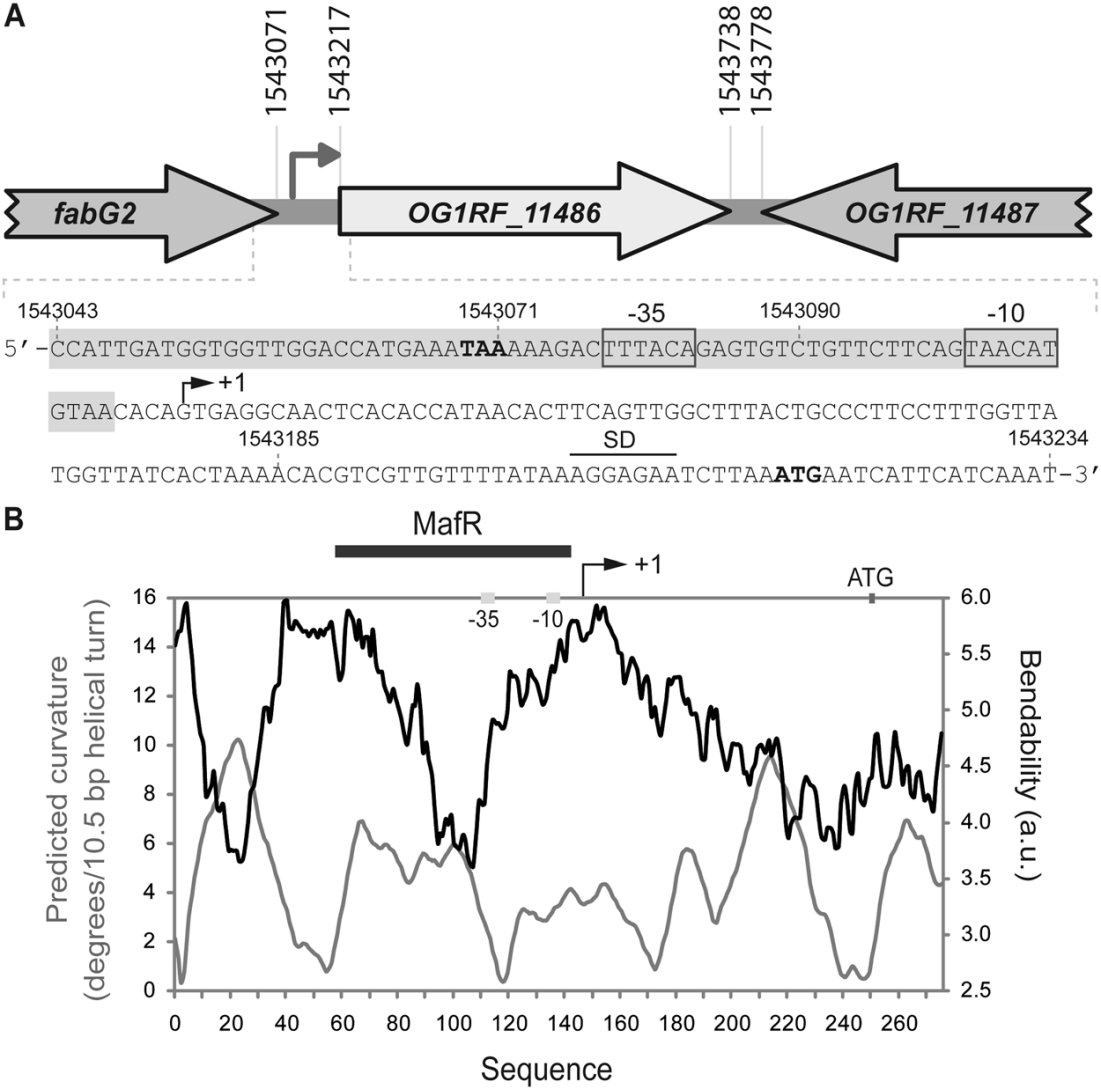
Relevant features of the *P11486* promoter region. (**A**) Genetic organization of the chromosome region that contains *OG1RF_11486*. Coordinates of the translation start and stop codons are indicated. The arrow upstream of the *OG1RF_11486* gene represents its promoter. The nucleotide sequence of the region spanning coordinates 1543043 to 1543234 is shown. The stop codon (TAA) of *fabG2* and the start codon (ATG) of *OG1RF_11486* are indicated in boldface letters. SD: Shine-Dalgarno sequence. The transcription start site (+1 position) of the *OG1RF_11486* gene, and the main sequence elements (−35 box and −10 box) of the *P11486* promoter identified in this work are indicated. The MafR binding site defined in this work is shown (shadowed box). Genes *OG1RF_11486* and *OG1RF_11487* correspond to genes *EF1774* and *EF1775* in *E. faecalis* strain V583. (**B**) Bendability/curvature propensity plot of the region spanning coordinates 1542969 to 1543243. The location of the *P11486* core promoter, the start codon of *OG1RF_11486* and the MafR binding site are indicated.

**Figure 5:**
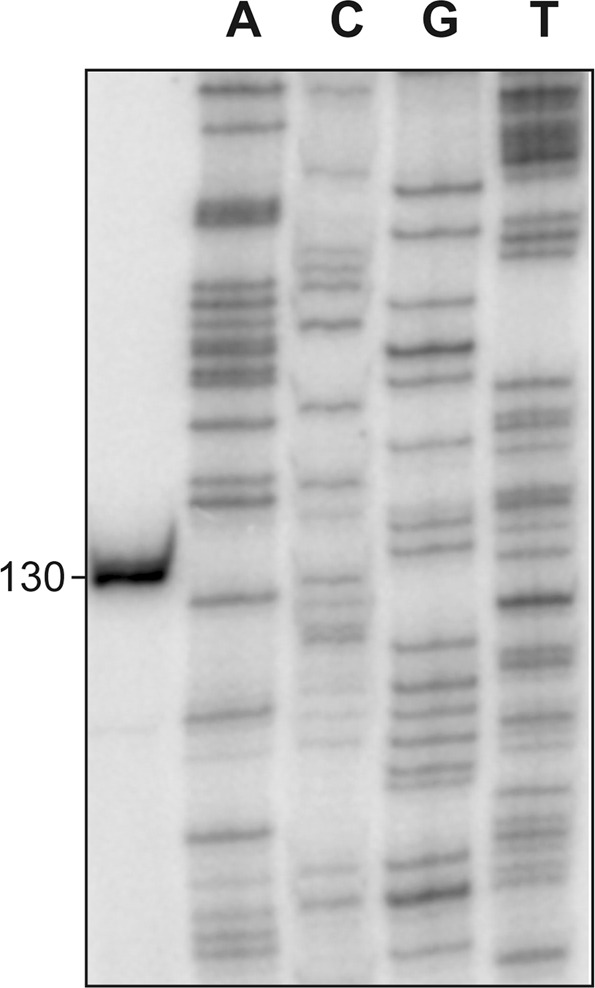
Transcription initiation site of the *OG1RF_11486* gene. Primer extension reactions were carried out using total RNA from OG1RF cells. Oligonucleotide R*11486*-D (coordinates 1543222-1543243) was used as primer. The size (in nucleotides) of the cDNA product is indicated on the left of the gel. Dideoxi-mediated chain termination sequencing reactions were used as DNA size markers (lanes A, C, G, T).

The BPROM program (*Softberry, Inc*.) predicts a promoter sequence (named *P11486* herein) upstream of the *OG1RF_11486* gene. The −35 (**TT**T**ACA**) and −10 (**TA**AC**AT**) elements of this promoter are separated by 17 nucleotides (Fig. 4A). By primer extension using total RNA from OG1RF cells, we demonstrated that the *P11486* promoter is functional *in vivo* (Fig. 5). Oligonucleotide R*11486*-D was used as primer (Table 1). A cDNA product of 130 nucleotides was detected, indicating that transcription of *OG1RF_11486* starts at coordinate 1543115 (Fig. 4A).

**Table 1:** Oligonucleotides used in this work.

Name	Sequence (5′ to 3′)^(a)^
F*mafR*-q	ACTTTATCAACCGTCCTTGG
R*mafR*-q	GTTTCGCCATAGACATTATC
F*zwf*-q	CGGTCAAGGGTTCAATACAACT
R*zwf*-q	CCAAGATTGGGCAACTTCGTCCCA
F*12294*-q	TCCTCTACCGTTGACACCTG
R*12294*-q	TGCCTTCGTTGACATCTCTTG
F*recA*-q	GCAACGAAATGGTGGAACAG
R*recA*-q	AAGGCATCGGCAATCTCTAAG
F*10600*-q	GCGTAGAAGAGTCAGCACTA
R*10600*-q	GCCATTCACAACGGTACAGC
F*11602*-q	CAACACCTCATTAGCGAAAC
R*11602*-q	GTCAATCATACCGACTAAACCA
F*11486*-q	TGGTTACCGCTTTGTATGTTG
R*11486*-q	CCCTAACGTAATGGACCAGAT
F*12294*	GAAACAGCGTTGA**G**CTCTTCTAGTGAC
R*12294*	CATTTCGTGTACCTCCGA**G**CTCCTTGATACCT
F*12294Δ69*	GTAAAAATGGTGAAA**G**A**GC**T**C**ATGTCAAAGCGT
F*12294Δ208*	CAGGGGCCACTGA**GC**T**C**ATCGACCATT
R*12294Δ-10*	CCTTTATTATAGAG**CTC**ATTTTCTTCACA
F*11486*	ACACCCATGAACG**G**AGCT**C**ATTTTGTA
R*11486*	ATAAAACAAC**G**AGCT**C**TTTTAGTGATAACC
F*11486Δ66*	GGGCCGTTG**A**GC**T**CAGCCACAGGAAGTA
F*11486Δ145*	GGCACAGTTATGAGC**T**CTGATGGTGGT
F*11486Δ169*	GTTGGACCATGAGCTCAAAAGACTTTACA
F*11486Δ188*	GACTTTACAGAG**CTC**CTGTTCTTCAGTA
F*12294*-D	GATGTCAAAGCGTTAATTGGCA
R*12294*-D	GACCCGTTTGCTTCGTCTTAGT
F*11486*-D	GCCACAGGAAGTAGCAAAACT
R*11486*-D	GGTTTGTGGATTTGATGAATGA

^(a)^Restriction sites are underlined, and the base changes that generate restriction sites are in bold.

**Figure 6:**
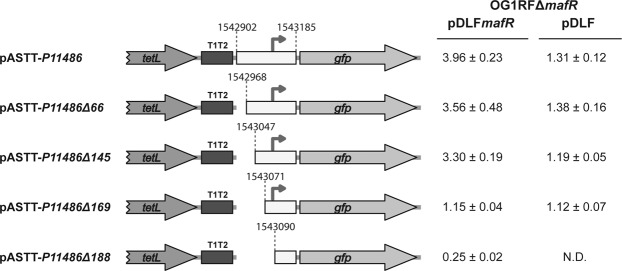
Effect of plasmid-encoded MafR on the activity of the *P11486* promoter. Five regions from the OG1RF chromosome were inserted independently into the *Sac*I site of pASTT. The coordinates of such regions are indicated. Gene *tetL*: tetracycline resistance determinant. Gene *gfp*: green fluorescent protein. The T1T2 box represents the tandem transcriptional terminators T1 and T2 of the *Escherichia coli rrnB* rRNA operon. The arrow represents the −35 element of the *P11486* promoter. The intensity of fluorescence (arbitrary units) corresponds to 0.8 ml of culture (OD_650_ = 0.4). In each case, three independent cultures were analysed. N.D.: non-determined.

To further characterize the *P11486* promoter, we constructed several transcriptional fusions (Fig. 6). A 284-bp DNA fragment (coordinates 1542902 to 1543185) was inserted into pASTT. The recombinant plasmid (pASTT-*P11486*) was first introduced into OG1RF and OG1RFΔ*mafR*. In both strains, *gfp* expression (1.48 ± 0.10 and 1.51 ± 0.16 units, respectively) was ∼4-fold higher than the basal level (OG1RF harbouring pASTT). This result indicated that the 284-bp DNA fragment has promoter activity, however, the chromosomal copy of *mafR* is not sufficient to activate such a promoter located on pASTT (multicopy plasmid). Next, we introduced pASTT-*P11486* into OG1RFΔ*mafR* harbouring pDLF*mafR* (plasmid-encoded MafR). In this strain, *gfp* expression was ∼3-fold higher than in the control strain (OG1RFΔ*mafR* harbouring pDLF) (Fig. 6). Similar results were obtained with plasmids pASTT-*P11486Δ*66 and pASTT-*P11486Δ145*, which allowed us to conclude that the 139-bp region between coordinates 1543047 and 1543185 contains both the *P11486* promoter and the site required for its activation by MafR. A further deletion analysis showed that sequences between coordinates 1543047 and 1543071 (plasmid pASTT-*P11486Δ169*) are needed for MafR-mediated activation of the *P11486* promoter but not for promoter activity. Moreover, deletion of the region that spans coordinates 1543071 and 1543090 (plasmid pASTT-*P11486Δ188*) removes the −35 element of the *P11486* promoter and, consequently, reduces the expression of *gfp* to basal levels (Fig. 6).

### MafR binds to the *P11486* promoter region *in vitro*

**Figure 7:**
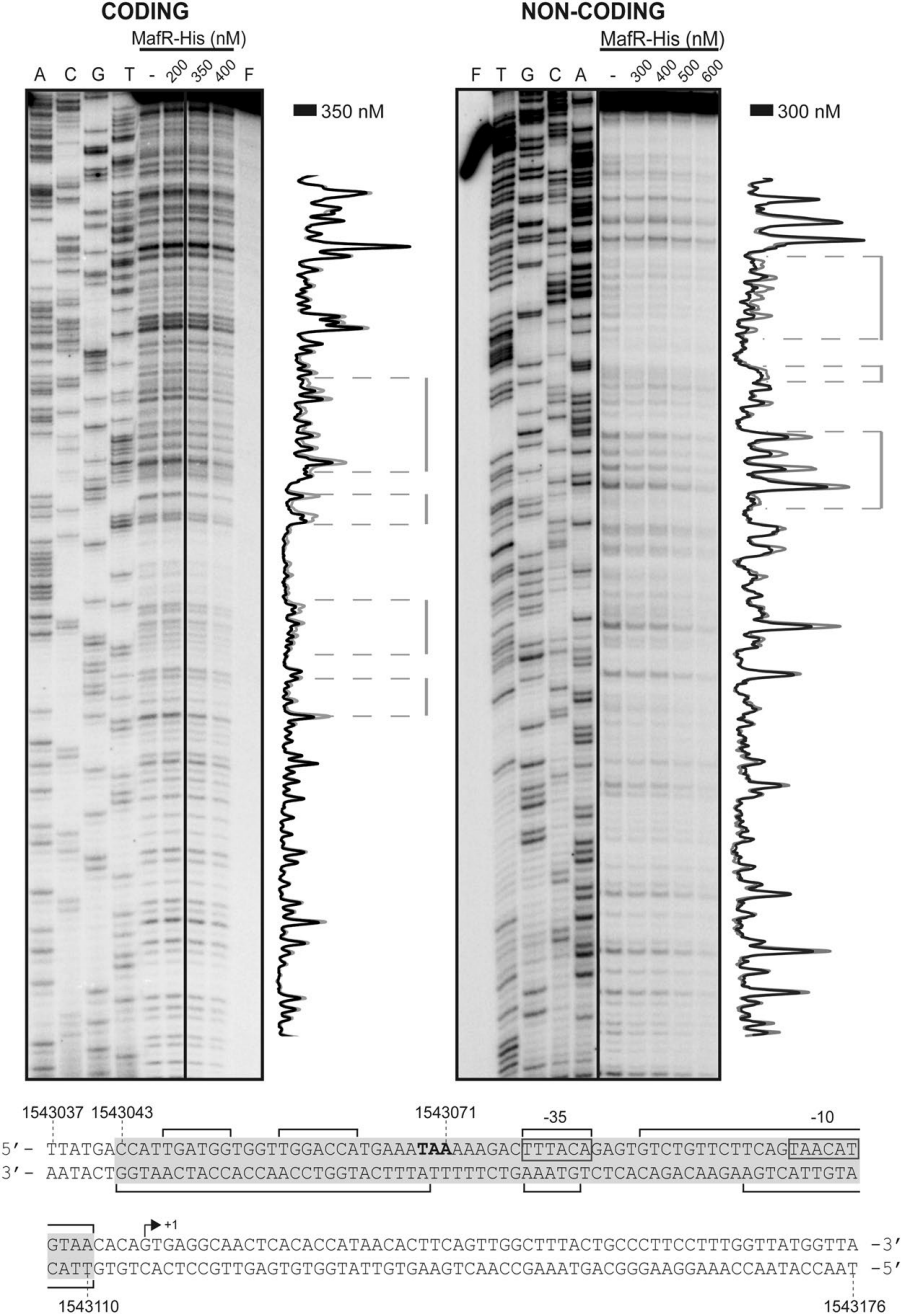
DNase I footprints of complexes formed by MafR-His on the 275-bp DNA fragment that contains the *P11486* promoter. ^32^P-labelled DNA (4 nM) was incubated with the indicated concentrations of MafR-His and then it was digested with DNase I. Non-digested DNA (F) and dideoxy-mediated chain termination sequencing reactions (A, C, G, T) were run in the same gel. All the lanes displayed came from the same gel (delineation with dividing lines). Densitometer scans corresponding to free DNA (grey line) and DNA with protein (black line) are shown. The nucleotide sequence of the region spanning coordinates 1543037 to 1543176 is shown. The transcription initiation site (+1 position) of *OG1RF_11486* is shown. The −35 and −10 elements of the *P11486* promoter are indicated. Brackets indicate regions protected against DNase I digestion. The site recognized by MafR-His (coordinates 1543043-1543110) is indicated with a grey box.

By DNase I footprinting assays, we analysed whether MafR-His binds to the *P11486* promoter region (Fig. 7). We used a 275-bp DNA fragment (coordinates 1542969 to 1543243), which contains both the *P11486* promoter and the site required for its activation by MafR *in vivo* (Fig. 6). On the coding strand and at 350 nM of MafR-His, changes in DNase I sensitivity (diminished cleavages) were observed within the region spanning coordinates 1543047 and 1543110. On the non-coding strand and at 300 nM of MafR-His, diminished cleavages were observed between coordinates 1543043 and 1543110. On both strands and at 400 nM of MafR-His, regions protected against DNase I digestion were observed along the DNA fragment, which is consistent with the ability of MafR-His to generate multimeric complexes^4^. Therefore, MafR-His recognizes preferentially a DNA site overlapping the *P11486* core promoter. Such a DNA site includes sequences needed for MafR-mediated activation of the *P11486* promoter *in vivo* (Fig. 6). According to the bendability/curvature propensity plot of the 275-bp DNA fragment, the MafR binding site contains regions of potential bendability (Fig. 4B).

## Discussion

Gene regulation plays a key role during bacterial adaptation to environmental fluctuations. The ability of enterococci to metabolize numerous carbohydrates enables them to colonize diverse environments^1^. Our previous work showed that MafR activates, directly or indirectly, the transcription of numerous genes on a genome-wide scale. Many of such genes encode proteins involved in transport or metabolism of carbon sources^5^. Now, by qRT-PCR, transcriptional fusions and DNase I footprinting experiments, we have demonstrated that MafR functions as a transcription activator. It activates directly the transcription of the *OG1RF*_*12294* and *OG1RF_11486* genes. Gene *OG1RF*_*12294* encodes a protein that has sequence similarity to several eukaryotic and prokaryotic proteins characterized as calcium P-type ATPases (Supplementary Table S1). This finding suggests that MafR could have a regulatory role in maintaining cellular calcium homeostasis. Calcium ions are known to affect different physiological processes in prokaryotic organisms, such as division, secretion, transport, and stress response^30^. Gene *OG1RF_11486* encodes a putative ECF transporter S-component, likely involved in the uptake of a queuosine precursor. Thus, MafR could have an additional regulatory role in the biosynthesis of queuosine, a modified nucleoside found at the wobble position of particular transfer RNAs^31^. There is evidence that queuosine contributes to the efficiency of protein synthesis. In *Shigella flexneri*, the intracellular concentration of the virulence-related transcriptional regulator VirF is reduced in the absence of queuosine^32^. Moreover, it has been reported that the lack of queuosine affects the growth of some bacteria under stress conditions^33,34^.

Bacteria use a variety of mechanisms to activate transcription from specific promoters. Genetic and biochemical studies have shown that some proteins stimulate transcription by binding to a specific DNA site either upstream of or overlapping the core promoter^35^. By DNase I footprinting experiments, we have found that MafR recognizes a site overlapping the *P12294* core promoter, as well as a site overlapping the *P11486* core promoter (this work). These results suggest that MafR might enhance the efficiency of both promoters by recruitment of RNA polymerase through direct interactions with the sigma factor. In addition, MafR might induce conformational changes in the target promoters, as it has been described for some transcription activators^35^. Transcriptional activation from specific promoters has also been reported for other members of the Mga/AtxA family. The pneumococcal Mga*Spn* regulator stimulates transcription of a four-gene operon (*spr1623-spr1626*) by binding to a specific DNA site upstream of the promoter (positions −60 to −99)^12^. Regarding the Mga regulator from *S. pyogenes*, the position of its DNA-binding site with respect to the start of transcription varies among the promoters tested. Nevertheless, the majority of the promoters contain an Mga binding site located around position −54, thereby overlapping the −35 element of the promoter^8^.

**Figure 8:**
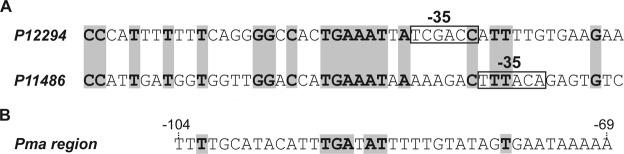
DNA sites recognized by MafR. (**A**) Nucleotide sequence alignment of the DNA sites recognized by MafR on the *P12294* and *P11486* promoter regions. Identical nucleotides are highlighted in grey boxes. (**B**) Nucleotide sequence of the DNA site recognized by MafR on the *Pma* promoter region (positions −69 to −104)^4^. Nucleotides shared with the MafR binding sites shown in (**A**) are highlighted in grey boxes.

Simple protein-DNA recognition mechanisms do not exist^36^. Based on the structures of various protein-DNA complexes, Rohs *et al*. proposed that particular proteins use likely a combination of readout mechanisms: base readout and shape readout^6^. The DNA sites recognized by MafR on the *P12294* and *P11486* promoters have a low sequence identity: they share the **GG**(C/A)**C**(A/C)(C/A)**TGAAAT**(T/A)**A** sequence element (Fig. 8A). Moreover, both MafR binding sites contain regions of potential bendability (Figs 1B and 4B). We have also shown that MafR recognizes a DNA site upstream of the *Pma* promoter (positions −69 to −104)^4^. The function of this interaction remains unknown. Such a MafR binding site is adjacent to the peak of a potential intrinsic curvature^4^ and shares a short DNA sequence motif (**TGA**T**AT**) with the two MafR binding sites identified in this work (Fig. 8B). Therefore, MafR does not seem to recognize a specific nucleotide sequence. Several findings suggest that recognition of particular DNA shapes could be a characteristic of the global regulators that constitute the Mga/AtxA family. Mga*Spn* from *S. pneumoniae* recognizes a DNA site upstream of the *P1623B* promoter (positions −60 to −99), as well as a DNA site overlapping the *Pmga* promoter (positions −23 to +21)^12^. The former interaction enhances the efficiency of the promoter^11^, whereas the function of the latter remains unknown. Such Mga*Spn* binding sites have a low sequence identity and, according to predictions, they contain an intrinsic curvature flanked by regions of bendability^12^. Furthermore, Mga*Spn* was shown to have a preference for AT-rich DNA regions^13^. Concerning Mga from *S. pyogenes*, several DNA-binding sites have been identified. These sites exhibit a low sequence identity (13.4%)^37^, although a consensus Mga binding sequence was initially proposed^38^. In the case of AtxA from *B. anthracis*, *in vitro* protein-DNA interaction studies have not been reported. Nevertheless, sequence similarities are not apparent in its target promoter regions, and some of them are intrinsically curved^39^.

In conclusion, our study shows for the first time that MafR is a transcription activator. It stimulates transcription from the *P12294* and *P11486* promoters *in vivo*. Moreover, MafR binds *in vitro* to a specific DNA site that overlaps the −35 element of each promoter. The two MafR binding sites have a low sequence identity but share a six-base pair motif. We propose that MafR would recognize intrinsic DNA structural features rather than particular DNA sequences on its target DNAs.

## Materials and Methods

### Oligonucleotides, bacterial strains, and plasmids

Oligonucleotides used in this work are listed in Table 1. *E. faecalis* strains OG1RF^14^ and OG1RFΔ*mafR*^5^ were used. Plasmids pDLF (expression vector) and pDLF*mafR* were described^5^. These plasmids carry a kanamycin resistance gene. Plasmid pASTT (D. García-Rincón, V. Solano-Collado and A. Bravo, unpublished results) is based on the pAST promoter-probe vector^40^, which carries a tetracycline resistance gene. Plasmid pASTT carries the *TrsiV* transcriptional terminator^40^ downstream of the *gfp* reporter gene. The following pASTT-derivatives were constructed in this work. In all cases, a region of the OG1RF chromosome was amplified by PCR using the indicated primers, digested with *Sac*I, and inserted into pASTT: pASTT-*P12294* (primers F*12294* and R*12294*, 260-bp restriction fragment), pASTT-*P12294Δ-10* (primers F*12294* and R*12294Δ-10*, 236-bp restriction fragment), pASTT-*P12294Δ69* (primers F*12294Δ69* and R*12294*, 192-bp restriction fragment), pASTT-*P12294Δ208* (primers F*12294Δ208* and R*12294*, 53-bp restriction fragment), pASTT-*P11486* (primers F*11486* and R*11486*, 290-bp restriction fragment), pASTT-*P11486Δ*66 (primers F*11486Δ66* and R*11486*, 224-bp restriction fragment), pASTT-*P11486Δ145* (primers F*11486Δ145* and R*11486*, 145-bp restriction fragment), pASTT-*P11486Δ169* (primers F*11486Δ169* and R*11486*, 121-bp restriction fragment), pASTT-*P11486Δ188* (primers F*11486Δ188* and R*11486*, 102-bp restriction fragment).

### Growth and transformation of bacteria

*E. faecalis* was grown in BHI medium, which was supplemented with tetracycline (4 μg/ml) and/or with kanamycin (250 μg/ml) when strains carrying plasmids were used. Experiments were performed at 37 °C without aeration. The protocol used to transform *E. faecalis* by electroporation was described^41^.

### DNA and RNA isolation

Genomic DNA was prepared using the Bacterial Genomic Isolation Kit (Norgen Biotek Corporation). Plasmid DNA was prepared using the High Pure Plasmid Isolation Kit (Roche Applied Science) as described^5^. Total RNA was isolated using the RNeasy mini Kit (QIAGEN). In general, bacteria were grown to an optical density at 650 nm (OD_650_) of 0.4 (logarithmic growth phase). For stationary phase, bacteria were grown to an OD_650_ of 0.8 and then incubated for two hours at the same temperature. Then, cultures were processed as reported^5^. The integrity of rRNAs was analysed by agarose gel electrophoresis. RNA concentration was determined using a NanoDrop ND-2000 Spectrophotometer.

### Polymerase chain reaction (PCR)

The Phusion High-Fidelity DNA polymerase (Thermo Scientific) and the Phusion HF buffer were used. Reaction mixtures (50 μl) contained 5–30 ng of template DNA, 20 pmol of each primer, 200 μM each deoxynucleoside triphosphate (dNTP), and one unit of DNA polymerase. PCR conditions were reported^40^. To amplify the 270-bp DNA fragment (promoter *P12294*) used in footprinting experiments, the Phusion GC buffer was used. In this case, reaction mixtures were supplemented with 7% DMSO and the annealing step was performed at 59 °C. PCR products were purified with the QIAquick PCR purification kit (QIAGEN).

### Quantitative RT-PCR (qRT-PCR)

For cDNA synthesis with random primers, the iScript Select cDNA Synthesis kit (Bio-Rad) was used as described^5^. Quantitative PCRs were performed using the iQ SYBR Green Supermix (Bio-Rad) and a iCycler Thermal Cycler (Bio-Rad) as reported^5^. Forward (F*gene*-q) and reverse (R*gene*-q) primers used in the quantitative PCRs are listed in Table 1. Relative quantification of gene expression was performed using the comparative *C*_T_ method^15^ as described^5^. Except for gene *mafR*, the internal control gene was *recA* (OG1RF_12439; recombination protein RecA). In the case of *mafR*, the internal control gene was *zwf* (OG1RF_10737; glucose-6-phosphate 1-dehydrogenase) because its expression level was similar at the logarithmic and stationary growth phases.

### Primer extension

Oligonucleotide R*11486*-D was radioactively labelled at the 5′-end using [γ-^32^P]-ATP (PerkinElmer) and T4 polynucleotide kinase (New England Biolabs) as reported^12^. Primer extension reactions (20 μl) contained 1.2 pmol of ^32^P-labelled oligonucleotide and 5 μg of total RNA isolated from strain OG1RF. The ThermoScript Reverse Transcriptase enzyme (Invitrogen) was used. Reactions were incubated at 55 °C for 45 min. After heating at 85 °C for 5 min, samples were ethanol precipitated and dissolved in loading buffer (80% formamide, 1 mM EDTA, 10 mM NaOH, 0.1% bromophenol blue, 0.1% xylene cyanol). cDNA products were analysed by sequencing gel (8 M urea, 6% polyacrylamide) electrophoresis. Dideoxy-mediated chain termination sequencing reactions were run in the same gel. Labelled products were visualized using a Fujifilm Image Analyser FLA-3000.

### Fluorescence assays

Plasmid-carrying cells were grown to an OD_650_ of 0.4 (logarithmic phase). Then, different volumes of culture (0.4 to 1 ml) were centrifuged, and cells were resuspended in 200 μl of phosphate-buffered saline (PBS). In each case, three independent cultures were analysed. Fluorescence intensity was measured using a Thermo Scientific Varioskan Flash instrument (excitation at 488 nm and emission at 515 nm). The fluorescence corresponding to 200 μl of PBS buffer without cells was ~0.03 arbitrary units.

### Purification of MafR-His

The procedure to overproduce and purify a His-tagged MafR_OG1RF_ protein (herein MafR-His) was reported^4^. MafR-His carries the Leu-Glu-6xHis peptide (His-tag) fused to its C terminus. Protein concentration was determined using a NanoDrop ND-2000 Spectrophotometer (Thermo Scientific).

### DNase I footprinting assays

Oligonucleotides were ^32^P-labelled at the 5′-end as described^12^. ^32^P-labelled oligonucleotides were used for PCR amplification to obtain double-stranded DNA fragments labelled at either the coding or the non-coding strand. Two regions of the OG1RF chromosome were amplified: a 270-bp region (coordinates 2425817-2425548) using the F*12294*-D and R*12294*-D oligonucleotides, and a 275-bp region (coordinates 1542969-1543243) using the F*11486*-D and R*11486*-D oligonucleotides. Binding reactions (8 μl) contained 30 mM Tris-HCl, pH 7.6, 1 mM DTT, 1 mg/ml BSA, 1.25% glycerol, 0.25 mM EDTA, 50 mM NaCl, 10 mM MgCl_2_, 1 mM CaCl_2_, 2–4 nM ^32^P-labelled DNA and different concentrations of MafR-His (100 to 600 nM). Reaction mixtures were incubated at room temperature for 20 min. Then, 0.015 units of DNase I (Roche Applied Science) was added and the reaction proceeded for 5 min at the same temperature. DNase I digestion was stopped by adding 1 μl of 250 mM EDTA. Then, 4 μl of loading buffer (80% formamide, 1 mM EDTA, 10 mM NaOH, 0.1% bromophenol blue and 0.1% xylene cyanol) was added. Samples were heated at 95 °C for 5 min and loaded onto sequencing gels (6% polyacrylamide, 8 M urea). Dideoxy-mediated chain termination sequencing reactions were run in the same gel. Labelled products were visualized using a Fujifilm Image Analyser FLA-3000. The intensity of the bands was quantified using the Quantity One software (Bio-Rad).

### *In silico* prediction of intrinsic curvature

The bendability/curvature propensity plots were calculated with the bend.it server^26^ (http://hydra.icgeb.trieste.it/dna/bend_it.html) as described previously^12^.

## Supplementary information

Supplementary Information
