# Supplementary material for: Transcriptional activation by MafR, a global regulator of Enterococcus faecalis
Source: Sci Rep. 2019 Apr 16;9:6146. doi: 10.1038/s41598-019-42484-4 (PMC6467988; doi:10.1038/s41598-019-42484-4)
Supplement: Supplementary file 1 — Supplementary Information [file 41598_2019_42484_MOESM1_ESM.doc]

**SUPPLEMENTARY INFORMATION**

**Transcriptional activation by MafR, a global regulator of *Enterococcus faecalis***

**Sofía Ruiz-Cruz, Ana Moreno-Blanco, Manuel Espinosa and Alicia Bravo***

Centro de Investigaciones Biológicas, Consejo Superior de Investigaciones Científicas, Madrid, Spain

***Correspondence:** Dr. Alicia Bravo**,** Centro de Investigaciones Biológicas**,** Consejo Superior de Investigaciones Científicas, Ramiro de Maeztu 9**,** E-28040 Madrid**,** Spain

Tel: +34 918373112

Fax: +34 915360432

Email: abravo@cib.csic.es

**Table S1.** Eukaryotic and prokaryotic proteins that have sequence homology to the enterococcal OG1RF_12294 protein (850 amino acids; GenBank AEA94981.1)

| **Protein**  **(Accession number)** | **Organism** | **Number of amino acids** | **Identity (%)** | **Similarity (%)** |
| --- | --- | --- | --- | --- |
| PMR1  (NP_011348.1) | *Saccharomyces cerevisiae*  S288C | 950 | 32.5 | 52.5 |
| PMR1  (CAC19896.1) | *Caenorhabditis elegans* | 901 | 33.0 | 52.2 |
| OG1RF_10600  (AEA93287.1) | *Enterococcus faecalis*  OG1RF | 881 | 36.0 | 53.1 |
| OG1RF_11602  (AEA94289.1) | *Enterococcus faecalis*  OG1RF | 901 | 39.7 | 56.9 |
| YloB  (NP_389448.1) | *Bacillus subtils*  168 | 890 | 36.7 | 56.4 |
| CaxP  (WP_000032453.1) | *Streptococcus pneumoniae*  D39 | 898 | 37.4 | 56.1 |
| LMCA1  (CAC98919.1) | *Listeria monocytogenes*  EGD-e | 880 | 35.9 | 56.6 |
| Lmo0818  (NP_464345.1) | *Listeria monocytogenes*  EGD-e | 876 | 34.8 | 53.4 |
| PMA1  (WP_010872526.1) | *Synechocystis sp.*  PCC 6803 | 905 | 36.3 | 53.5 |

Accession numbers were obtained from NCBI (National Center for Biotechnology Information).

Identity and similarity are given according to EMBOSS Needle (Pairwise Sequence Alignment) (Rice, P., Longden, I., Bleasby, A. 2000, EMBOSS: the European Molecular Biology Open Software Suite, *Trends Genet*.,**16**, 276-277).

**Table S2.** Putative metal-ion P-type ATPases encoded by the *E. faecalis* OG1RF genome

| **Locus tag** | **Description** |
| --- | --- |
| OG1RF_10241 | Copper-exporting ATPase |
| OG1RF_10242 | MerTP family copper permease, binding protein CopZ |
| OG1RF_10493 | P-type ATPase cadmium transporter |
| OG1RF_10600 | Putative calcium-transporting ATPase |
| OG1RF_10603 | P-type ATPase copper (Cu) transporter |
| OG1RF_11036 | E1-E2 family cation-transporting ATPase |
| OG1RF_11074 | Magnesium-importing ATPase |
| OG1RF_11140 | Magnesium-importing ATPase |
| OG1RF_11189 | Zinc-exporting ATPase |
| OG1RF_11240 | E1-E2 family cation-transporting ATPase |
| OG1RF_11602 | Putative calcium-transporting ATPase |
| OG1RF_11999 | P-type ATPase cadmium transporter |
| OG1RF_12294 | P-type ATPase cation transporter |
